# SPARC overexpression in primary tumors correlates with disease recurrence and overall survival in patients with triple negative breast cancer

**DOI:** 10.18632/oncotarget.10532

**Published:** 2016-07-11

**Authors:** Anjie Zhu, Peng Yuan, Feng Du, Ruoxi Hong, Xiaoyan Ding, Xiuqing Shi, Ying Fan, Jiayu Wang, Yang Luo, Fei Ma, Pin Zhang, Qing Li, Binghe Xu

**Affiliations:** ^1^ Department of Medical Oncology, National Cancer Center/Cancer Hospital, Chinese Academy of Medical Sciences and Peking Union Medical College(CAMS&PUMC), Beijing, China; ^2^ Department of Medical Oncology, Sun Yat-sen University Cancer center, The State Key Laboratory of Oncology in South China, Collaborative Innovation Center for Cancer Medicine, Guangzhou, Guangdong, China; ^3^ Department of Medical Oncology, Beijing Ditan Hospital, Capital Medical University, Beijing, China; ^4^ Department of Medical Oncology, Yuhuangding Hospital, Yantai, Shandong, China

**Keywords:** SPARC, osteonectin, triple-negative breast cancer, prognosis

## Abstract

SPARC/osteonectin expression is reportedly altered in various malignancies. However, little is known regarding to the prognostic value of SPARC in triple-negative breast cancer (TNBC) patients. In this study, immunohistochemistry and immunoreactive scores (IRSs) were used to evaluate SPARC protein expression in primary tumors from 211 TNBC patients with up to 10 years of clinical follow-up data. High SPARC expression (IRS ≥3) was detected in 52.1% of primary tumors. Patients expressing high SPARC levels had worse disease-free survival (DFS) (HR=1.58, 95% CI: 1.01-2.47, P=0.044) and overall survival (OS) (HR=1.74, 95% CI: 1.06-2.85, P=0.029) than patients with lower SPARC levels. Furthermore, high SPARC expression was an independent prognostic factor for both DFS (HR=1.73, 95% CI: 1.10-2.73, P=0.018) and OS (HR=1.90, 95% CI: 1.14-3.16, P=0.014) in TNBC patients. These results suggest that increased SPARC expression may be an indicator of greater aggressiveness, and may serve as a prognostic factor for triple-negative breast cancer.

## INTRODUCTION

Breast cancer is the most common malignant tumor in women. China contributes 12.2% of all newly diagnosed breast cancer cases and 9.6% of breast cancer related deaths worldwide [1]. Accounting for 10-17% of all breast carcinomas, triple-negative breast cancer (TNBC), which lacks the expression of estrogen receptor (ER), progesterone receptor (PR) and HER2, is the most aggressive subtype and is known for its poor prognosis and high recurrence probability [2]. TNBC tumors are often larger at presentation and possess more advanced histologic grade compared to ER/PR positive breast cancers [3]. Biomarkers for TNBC prognosis are currently ill defined, making it the true challenge to the modern oncology.

SPARC (secreted protein acidic and rich in cysteine), also known as osteonectin or BM-40, is an albumin-binding glycoprotein, which is secreted by cells and modulates their interactions with extracellular matrix [4]. SPARC plays a crucial role in the regulation of cellular functions, such as proliferation and cell migration [5]. Although SPARC is not a tumor-specific protein, its overexpression is associated with tumor growth, metastasis, and aggressiveness [6, 7]. Importantly, since SPARC binds albumin with a high affinity [8], high SPARC tumor levels could enhance the accumulation of albumin within the tumor tissue, and improve the response to nanoparticle albumin-bound (nab)-paclitaxel in a targeted way [9].

An increasing number of studies have shown altered SPARC expression in various types of cancer. Overexpression of SPARC correlates with poor prognosis in pancreatic cancer [10]. However, the role of SPARC in breast cancer development and progression is controversial. Several studies have suggested that SPARC expression is higher in TNBC than in other breast cancer subtypes, and correlates with poor prognosis [11-13], while other studies have shown nonsignificant or opposite results [14-17]. Similar conflicting findings have been reported in the TNBC subgroup alone [13, 14, 17]. The small size of cohorts, different approaches of evaluation, and the lack of standard assessment methodology may contribute to these contradictory findings.

The current retrospective study has been designed to investigate the SPARC expression in human TNBC tissues and to assess its potential prognostic value. With up to 10-year clinical follow-up, we report here that the high SPARC expression is an independent prognostic factor for recurrence and death in patients with TNBC after adjusting for factors, such as age, menopausal status, histopathologic grade, tumor size, lymph node metastasis, vascular invasion, tumor staging, and cancer therapies.

## RESULTS

### Basic characteristics and SPARC expression

SPARC protein levels were analyzed by IHC of 211 TNBC specimens. SPARC protein was found predominantly in the cytoplasm of tumor cells, and 52.1% cases showed high cytoplasmic staining. Stromal staining was observed in almost all samples. Only few cases exhibited nuclear SPARC localization.

There were 110 patients in the high SPARC group (IRS ≥3) and 101 patients in the low SPARC group (IRS < 3). There was no significant correlation between cytoplasmic SPARC localization and clinicopathological parameters, including age, menopausal status, histopathologic grade, tumor size, lymph node metastasis, vascular invasion, or TNM staging (Table 1).

**Table 1:** Patient characteristics and association of SPARC expression with clinicopathological data

	All Cases	SPARC Low	SPARC High	P-value*
**All patients**	211(100)	101 (47.87)	110 (52.13)	
**Age (year)**				
< 60	162 (76.78)	78 (48.15)	84 (51.85)	0.882
≥ 60	49 (23.22)	23 (46.94)	26 (53.06)	
**Menopausal status at diagnosis**				
Premenopausal	109 (51.66)	49 (44.95)	60 (55.05)	0.381
Postmenopausal	102 (48.34)	52 (50.98)	50 (49.02)	
**The histopathologic grading**				
I-II	83 (39.34)	40 (48.19)	43 (51.81)	0.939
III	128 (60.66)	61 (47.66)	67 (52.34)	
**Tumor size(cm)**				
≤ 2.0	74 (35.07)	37 (50)	37 (50)	0.649
> 2.0	137 (64.93)	64 (46.72)	73 (53.28)	
**Lymph node metastasis**				
No	103 (48.82)	51 (49.51)	52 (50.49)	0.64
Yes	108 (51.18)	50 (46.3)	58 (53.7)	
**TNM staging**				
I-II	144 (68.25)	68 (47.22)	76 (52.78)	0.783
III	67 (31.75)	33 (49.25)	34 (50.75)	
**Vascular Invasion**				
No	177 (83.89)	85 (48.02)	92 (51.98)	0.918
Yes	34 (16.11)	16 (47.06)	18 (52.94)	

* The parametric p-value is calculated by chi-square test.

### Survival analysis

Univariable and multivariable logistic regression analyses revealed that SPARC protein levels correlated with both DFS (Table 2) and OS (Table 3). Univariable analysis demonstrated that patients with high SPARC expression had worse 5-year DFS (56% vs 71.2%, HR=1.58, 95% CI: 1.01-2.47, P = 0.044) and 5-year OS (71.8% vs 81.1%, HR=1.74, 95% CI: 1.06-2.85, P = 0.029) compared to those with low SPARC protein levels. In multivariable analysis, high SPARC expression (HR=1.73, 95% CI: 1.10-2.73, P=0.018), large tumor size (P=0.012), lymph node metastasis (P=0.002), vascular invasion (P=0.040), and chemotherapy without paclitaxel (PTX) (P=0.018) were independently predictive for DFS in all patients. In addition, for overall survival, high SPARC expression (HR=1.90, 95%CI: 1.14-3.16, P=0.014), large tumor size (P=0.039) and lymph node metastasis (P=0.001) were independent risk factors.

**Table 2:** Univariable and multivariable analysis for of TNBC disease-free survival

	Univariable analysis		Multivariable analysis	
	HR (95% CI)	P-value	HR (95% CI)	P-value
**SPARC**	1.58 (1.01-2.47)	0.044	1.73 (1.10-2.73)	0.018
**Age**	1.21 (0.74-1.99)	0.454	1.61 (0.82-3.15)	0.166
**Menopausal status**	0.86 (0.56-1.34)	0.512	0.69 (0.38-1.23)	0.209
**Histopathologic grade**	0.82 (0.53-1.28)	0.384	0.86 (0.55-1.36)	0.525
**Tumor size**	2.43 (1.42-4.15)	0.001	2.07 (1.17-3.65)	0.012
**LNM**	3.27 (2.02-5.31)	< 0.001	2.77 (1.47-5.24)	0.002
**TNM staging**	2.79 (1.80-4.32)	< 0.001	1.30 (0.71-2.37)	0.401
**Vascular Invasion**	2.12 (1.28-3.52)	0.004	1.77 (1.03-3.04)	0.040
**Chemotherapy with/without PTX**	1.06 (0.68-1.65)	0.803	0.54 (0.32-0.90)	0.018
**Radiation therapy**	2.17 (1.40-3.37)	< 0.001	1.21 (0.68-2.15)	0.519

HR, hazard ratio; CI, confidence interval

**Table 3:** Univariable and multivariable analysis for of TNBC overall survival

	Univariable analysis		Multivariable analysis	
	HR (95% CI)	P-value	HR (95% CI)	P-value
**SPARC**	1.74 (1.06-2.85)	0.029	1.90 (1.14-3.16)	0.014
**Age**	1.37 (0.81-2.33)	0.245	2.19 (0.99-4.84)	0.052
**Menopausal status**	0.83 (0.51-1.35)	0.460	0.65 (0.32-1.33)	0.234
**Histopathologic grade**	0.87 (0.54-1.42)	0.583	0.84 (0.50-1.39)	0.490
**Tumor size**	2.69 (1.47-4.93)	0.001	1.96 (1.04-3.72)	0.039
**LNM**	4.88 (2.70-8.81)	< 0.001	3.37 (1.60-7.11)	0.001
**TNM staging**	3.80 (2.34-6.17)	< 0.001	1.42 (0.74-2.71)	0.290
**Vascular Invasion**	2.24 (1.31-3.86)	0.003	1.56 (0.88-2.75)	0.126
**Chemotherapy with/without PTX**	1.21 (0.74-1.98)	0.456	0.58 (0.33-1.03)	0.064
**Radiation therapy**	2.84 (1.73-4.65)	< 0.001	1.55 (0.83-2.88)	0.169

HR, hazard ratio; CI, confidence interval

## DISCUSSION

To our knowledge, this is the largest analysis that addresses the association between SPARC protein expression and the survival rates in TNBC patients. In addition, our study includes up to 133.0 months of follow-up data. This study indicates that high SPARC expression contributes to TNBC progression, and may serve as an independent prognostic factor for the disease outcome.

SPARC may play an important role in the progression of breast cancer. SPARC expression was increased in 37% of TNBC tumor tissues (P = 0.038) compared with other breast cancer subtypes [14]. In the neoadjuvant GeparTrio trial, 667 patients were evaluated for SPARC expression by immunohistochemistry using a standardized immunoreactive score (IRS). The study demonstrated that high SPARC expression (IRS ≥ 6) was associated with an increased pathological complete response (pCR) compared with low SPARC expression (IRS < 6) (47% vs. 26%, P = 0.032) [14]. SPARC was an independent predictive factor in the overall population (P = 0.010) as well as in the TNBC subgroup (P = 0.036). However, no statistically significant association of SPARC expression with overall survival and progression-free survival was observed in that study [14].

Although SPARC controls important mechanisms in tumor development and progression, its actual function is still contradictory and not fully understood. For example, in prostate cancer, SPARC may function as a tumor suppressor since down-regulation or inactivation of SPARC enhances aggressive and metastatic behavior [18]. On the other hand, in colorectal cancer, SPARC was suggested to have a pro-tumorigenic and pro-metastatic function [19]. There is little evidence of SPARC's effect in breast cancer patients, especially in TNBC. Several studies have demonstrated that high SPARC expression is associated with small tumor size, low histological grade, and high ER expression (all P < 0.0001), which are all features of slow growing and low proliferating tumors [13, 17]. However, consistent with the GeparTrio study [14], we have found that high SPARC expression is not associated with tumor size or histopathologic grade. Nagai et al also showed that SPARC protein expression is not associated with lymph node metastasis in breast cancer [17]; this was confirmed by Beck et al, and Bergamaschi et al [20, 21]. Importantly, Azim et al evaluated SPARC mRNA levels and demonstrated that high SPARC gene expression was associated with poor clinical outcome [13]. Therefore, the SPARC function may be context dependent [22]. More studies are needed to explore the exact mechanism by which SPARC regulates tumor development and progression.

With 10-year follow-up, Nagai et al [17] reported that SPARC down-regulation correlated with poor prognosis in patients with TNBC. They found that patients with tumors classified as triple negative had the worst 10-year cumulative survival rate (40.3%, P < 0.001). However, their study suggested that patients expressing low SPARC protein levels had worse DFS (P = 0.001) and OS (P = 0.001) compared with those with high SPARC levels. They concluded that the prognosis was worse when the tumor showed negative SPARC immunostaining. The difference to our study is the evaluating approaches and definitions for SPARC positivity and the cutoff value for high expression of SPARC. While Nagai et al [17] analyzed SPARC expression quantitatively using the image capture system, we evaluated the staining intensity and proportion of positive cells and integrated both parameters in a semi-quantitative IRS, which is applicable for broad routine clinical practice [23].

In cancer patients, high SPARC protein levels may enhance the tumor concentration of nab-paclitaxel and improve the treatment response. Nab-paclitaxel is approved as a second-line treatment in advanced breast cancer. The GeparSepto-GBG 69 study revealed that nab-paclitaxel significantly increased the rate of patients achieving a pCR response after anthracycline-based chemotherapy, especially in TNBC patients [24]. One of the subgroups was stratified according to cytoplasmic SPARC protein levels (positive [IRS of 6-12] vs negative [IRS 0-5]) in tumor tissues. They found that the pCR rate was significantly higher in the nab-paclitaxel group than solvent-based paclitaxel group (38% vs 29%, P = 0.0034) in SPARC-negative group. However, in the SPARC-positive group, the pCR rate was similar between groups (42% vs 30%, p = 0.10). Furthermore, in patients treated with nab-paclitaxel, pCR rate was also comparable between the SPARC-negative and positive groups (38% vs 42%). However, in the GeparTrio trial [14], increased SPARC level (IRS ≥6) was associated with a markedly higher pCR rate compared with low SPARC level (IRS < 6). This inconsistency may be attributed, in part, to the patients enrolled. The rate of SPARC-positivity was lower in the GeparSepto-GBG 69 study than in the GeparTrio trial (15.8% vs 26%). Additionally, only 23% patients had TNBC. Therefore, further studies should investigate the potential use of SPARC as a predictive biomarker of responsiveness to nab-paclitaxel therapy in TNBC as well as in other types of breast cancer.

A relative weakness of our study is that the sample size is still relatively small despite the fact that it is the largest cohort so far. Another noteworthy point is that we observed stromal staining in several cases, which might pose an interpretation problem in the studies evaluating SPARC expression using digital imaging. In this regard, future studies should analyze and correlate the SPARC protein levels in stroma with the cytoplasmic SPARC levels and TNBC prognosis.

## CONCLUSION

Our findings indicate that SPARC could serve as a useful prognostic biomarker in TNBC. Data from large prospective studies in TNBC as well as other types of breast cancer are warranted. Further studies focusing on the association between SPARC expression and the benefit of nab-paclitaxel therapy are urgently needed. In addition, it would be beneficial to develop a consensus on the evaluation of SPARC expression. Ideally, SPARC expression should be applicable in broad routine clinical practice to provide information of disease prognosis and treatment response.

## MATERIALS AND METHODS

### Study design and patient characteristics

Primary breast tumor samples were obtained from 211 women who were pathologically diagnosed with TNBC in Cancer Hospital, Chinese Academy of Medical Sciences between 2004 and 2008. All patients were treated with primary radical mastectomy, modified radical mastectomy, or breast-conserving surgery. Patients received adjuvant (before or after surgery) chemotherapy with or without paclitaxel. None of the patients received radiotherapy before mastectomy.

The mean age of patients at the time of diagnosis was 50.9 years (range 21 - 83 years). Clinicopathological data, including age, menopausal status, histopathologic grade, tumor size, lymph node metastasis, vascular invasion, tumor staging (TNM), and survival data were obtained from medical records. The median follow-up period was 90.5 months (range 8.2 – 133.0 months). TNM staging was classified based on the criteria for breast cancer of the American Joint Committee on Cancer. Table 1 summarizes the detailed clinicopathological parameters.

All patients gave written informed consent permitting the use of their breast tissue for research at the time the specimens were obtained. The research protocol was reviewed and approved by the Ethical Committee and Institutional Review Board of Cancer Hospital, Chinese Academy of Medical Sciences.

### Immunohistochemical staining of SPARC

**Figure 1:**
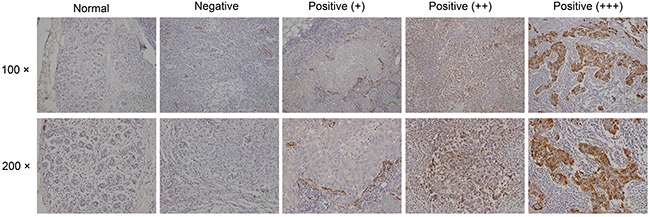
Immunohistological SPARC expression in tumors and normal breast tissue

All samples taken from surgery were fixed with formalin and embedded in paraffin. Tumor tissues were stained with H&E. Immunohistochemical staining of SPARC (using mouse anti-osteonectin/SPARC antibody diluted 1:200; Novex by Life Technologies) was carried out according to manufacturer's recommendation. The staining intensity (negative = 0, weak = 1, moderate = 2, strong = 3) and percentage of tumor positive cells (0%=0, 1% - 10%=1, 11% - 50%=2, 51% - 80%=3, 81% -100%=4) were evaluated (Figure 1). Immunoreactive scores (IRS) ranging from 0 to 12 were calculated by multiplying the numeric values of both parameters [14, 25].

Based on data distribution, the cases were divided into two groups with low or high cytoplasmic SPARC expression (IRS <3 versus IRS ≥3); this cutoff point was defined by the web-based software Cutoff Finder (http://molpath.charite.de/cutoff/) using the significance of correlation with survival variable method [26].

### Statistical analysis

SAS version 9.4 software (SAS Institute, Gray, NC) was used for statistical analysis. Fisher exact tests and Pearson χ2 tests were used to explore the correlation between SPARC expression and clinicopathological characteristics. Hazard ratios (HRs) and 95% confidence intervals (CIs) were calculated using multivariable Cox proportional hazards models. The proportionality assumption was examined using models that allowed time-dependent HRs and no evidence was found that HRs varied with time. All P values are two-sided and P values less than 0.05 were considered statistically significant.
